# Microplastics ingestion and heterotrophy in thermally stressed corals

**DOI:** 10.1038/s41598-019-54698-7

**Published:** 2019-12-03

**Authors:** Jeremy B. Axworthy, Jacqueline L. Padilla-Gamiño

**Affiliations:** School of Aquatic and Fishery Sciences, University of Washington, Seattle, Washington 98195 USA

**Keywords:** Ecophysiology, Physiology

## Abstract

Rising sea temperatures and increasing pollution threaten the fate of coral reefs and millions of people who depend on them. Some reef-building corals respond to thermal stress and subsequent bleaching with increases in heterotrophy, which may increase the risk of ingesting microplastics. Whether this heterotrophic plasticity affects microplastics ingestion or whether ingesting microplastics affects heterotrophic feeding in corals is unknown. To determine this, two coral species, *Montipora capitata* and *Pocillopora damicornis*, were exposed to ambient (~27 °C) and increased (~30 °C) temperature and then fed microplastics, *Artemia* nauplii, or both. Following thermal stress, both species significantly reduced feeding on *Artemia* but no significant decrease in microplastics ingestion was observed. Interestingly, *P*. *damicornis* only ingested microplastics when *Artemia* were also present, providing evidence that microplastics are not selectively ingested by this species and are only incidentally ingested when food is available. As the first study to examine microplastics ingestion following thermal stress in corals, our results highlight the variability in the risk of microplastics ingestion among species and the importance of considering multiple drivers to project how corals will be affected by global change.

## Introduction

Reef building corals (Scleractinia) are increasingly challenged by a suite of anthropogenic stressors including pollution and rising sea temperatures due to climate change^1,2^. These stressors threaten the fate of coral reefs and the ecosystem services they provide which support the livelihoods of tens of millions of people worldwide^3^. Model projections forecast that more than 75% of coral reefs will be subjected to annual severe bleaching before 2070 due to thermal stress alone^4^, but the fate of corals may be worsened when they face additional stressors^5,6^. Recent evidence suggests that microplastics (plastic particles or fibers <5 mm), may negatively affect corals^7–10^. To date, however, no studies have looked at the potential for thermal stress to affect microplastics ingestion by reef-building corals.

Under normal conditions, most reef-building corals acquire the majority of their energy from a symbiotic partnership with photosynthetic dinoflagellates in the family Symbiodiniaceae^11^, while less energy is generally derived from heterotrophic feeding on zooplankton^12–14^. When thermally stressed, Symbiodiniaceae are expelled from corals (bleaching) leading to a net decrease in autotrophic energy acquisition^15,16^. If elevated temperatures persist, corals deplete their energy reserves and can starve, but if the temperature reduces before the corals’ energy reserves are exhausted, Symbiodiniaceae can be reacquired and the coral may recover^17–19^.

Some corals respond to thermal stress and subsequent bleaching by increased heterotrophy which shifts the corals’ reliance from energy derived from photosynthesis to energy derived from zooplankton prey, an adaptation termed heterotrophic plasticity^14,20–23^. While the underlying mechanisms and timing of this response are still unclear, increased carbon acquisition from heterotrophy can help corals maintain daily metabolic costs until Symbiodiniaceae can be reacquired. In contrast, other corals decrease their feeding rate during, or following, thermal stress^22–24^ which may negatively impact their resilience. For corals that display heterotrophic plasticity, increased feeding of zooplankton prey could potentially increase their risk of ingesting unwanted particles in the water, such as microplastics.

Microplastics are considered ubiquitous in aquatic ecosystems worldwide and are negatively impacting marine life^25^. By 2014, there was an estimated 15 to 51 trillion microplastic particles in the oceans^26^, which are derived from direct manufacturing or break down from larger plastic debris due to abrasion, wave action, and UV radiation. Plastic waste entering the oceans is expected to increase 10-fold by 2025^27^ leading to growing concerns about the potential for these pollutants to negatively affect marine organisms. Their similarity in shape and size to zooplankton make microplastics particularly problematic for planktivorous animals such as corals that can ingest them while feeding^8^. In some organisms, ingesting microplastics can lead to decreased feeding efficiency, growth and fecundity^9,28,29^ but for corals these effects are still not fully understood. Further, there is increasing concern about the role of plastics, large and small, to act as vectors for diseases and contaminants^30–32^.

Previous studies have demonstrated that ingesting, and exposure to, microplastics can have negative effects on corals. Corals that ingested microplastics tended to egest most of them within 48 h which limited the time microplastics could cause internal damage but is still thought to be energetically costly^7–9^. For some coral species, exposure to microplastics resulted in increased mucous production, bleaching, necrosis, changes in photosynthetic performance, and decreased growth and feeding rates^7,9,10^. One coral species, *Astrangia poculata*, appeared to selectively feed on clean microplastics when also offered bio-fouled particles, leading researchers to suggest that chemical cues released by plastics (i.e., chemoreception) drove ingestion^33^. Additional research also showed that *A*. *poculata* preferred to feed on microplastics over similar sized brine shrimp eggs, and that ingesting microplastics can inhibit later feeding on nutritious prey^32^. While we are beginning to understand the responses and mechanisms of microplastics ingestion by corals, we still do not know how this pervasive pollutant interacts with other stressors, such as rising sea temperatures.

The objective of this study was to examine whether prior exposure to thermal stress affects microplastics ingestion and if microplastics exposure and ingestion affects the amount of prey ingested by reef-building corals. To determine this, we compared ingestion rates of corals exposed to microplastics (MP) only, *Artemia* only, or MP and *Artemia* following ambient and increased temperature treatments. We hypothesized that if *Artemia* ingestion changed due to thermal stress, we would also see a similar trend in MP ingestion rates. Additionally, if a chemical in microplastics makes them more appealing to corals^33^, then we hypothesized that corals exposed to microplastics would ingest less prey in favor of microplastics. As thermal stress events are predicted to occur with greater frequency and intensity, and microplastics continue to accumulate in the oceans, it is critical that we understand how corals respond to these stressors to better manage coral resilience in our changing world.

## Results

### Microplastics ingestion

**Figure 1:**
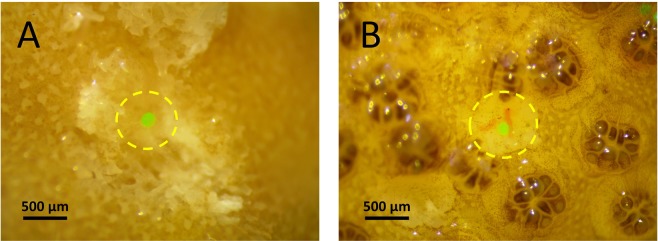
Microplastics ingested in the polyps of (**A**) *Montipora capitata*, and (**B**) *Pocillopora damicornis*. The yellow dotted circles show where the polyp was dissected exposing the contents of the gut.

Both species, *Montipora capitata* and *Pocillopora damicornis*, ingested microplastics (Fig. 1). The number of microplastics ingested by individual polyps ranged from zero to one in *M*. *capitata*, and from zero to seven in *P*. *damicornis*. Overall, *P*. *damicornis* ingested over 520% more microplastics than *M*. *capitata*.

**Figure 2:**
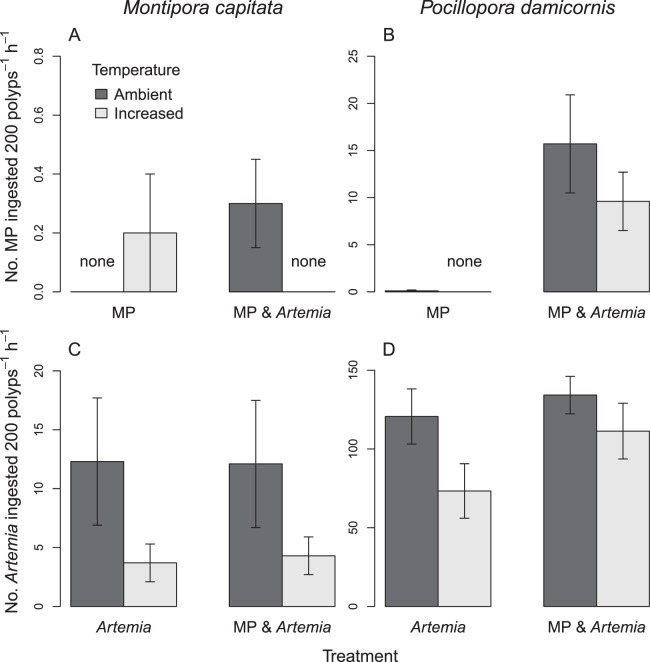
Mean ( ± SEM) microplastics ([MP], **A**,**B**) and *Artemia* nauplii (**C**,**D**) ingestion rates of corals exposed to ambient (dark bars) and increased (light bars) temperature. Note the difference in scale of the y-axes.

Compared to ambient temperature controls, corals exposed to three weeks of thermal stress were visibly pale (bleached). This did not, however, result in significantly different microplastics ingestion rates (Fig. 2A,B). *M*. *capitata*, ingested very few microplastics overall, ingesting only 0.2 ± 0.2 (mean ± 1 SEM) microplastics per 200 polyps h^−1^ in the MP only treatment after thermal stress, and 0.3 ± 0.2 microplastics per 200 polyps h^−1^ in the MP & *Artemia* treatment at ambient temperature (Fig. 2A). *M*. *capitata* did not ingest any microplastics in the MP only treatment at ambient temperature or in the MP & *Artemia* treatment after thermal stress (Fig. 2A).

Though thermal stress was not a significant factor, *P*. *damicornis* ingested significantly more microplastics in the MP & *Artemia* treatments than in the MP only treatments after both temperature treatments (Permutation ANOVA [aovp], df = 1, F = 20.16, p = 0.00012, Fig. 2B). In the MP & *Artemia* treatments *P*. *damicornis* ingested 15.7 ± 5.2 microplastics per 200 polyps h^−1^ at ambient temperature and 9.6 ± 3.1 microplastics per 200 polyps h^−1^ after thermal stress (Fig. 2B). When *P*. *damicornis* was exposed to microplastics only, particle ingestion was absent or negligible after both ambient and increased temperature treatments (Fig. 2B).

### *Artemia* ingestion

*Artemia* were ingested by both species in all treatments (Fig. 2C,D). The number of *Artemia* ingested by individual polyps ranged from zero to one in *M*. *capitata* and zero to twelve in *P*. *damicornis*.

Following thermal stress, *Artemia* ingestion was significantly decreased in both *M*. *capitata* (aovp, df = 1, F = 11.65, p = 0.002, Fig. 2C) and *P*. *damicornis* (aovp, df = 1, F = 6.658, p = 0.0156, Fig. 2D) compared to ambient temperature controls. For both species, there was no significant difference in *Artemia* ingestion between MP only and MP & *Artemia* treatments after either temperature treatment. *M*. *capitata* in the *Artemia* only treatments ingested 12.3 ± 5.4 *Artemia* per 200 polyps h^−1^ at ambient temperature and 3.7 ± 1.6 *Artemia* per 200 polyps h^−1^ after thermal stress. In the MP & *Artemia* treatments, *M*. *capitata* ingested 12.1 ± 5.4 *Artemia* per 200 polyps h^−1^ at ambient temperature and 4.3 ± 1.6 *Artemia* per 200 polyps h^−1^ after thermal stress (Fig. 2C). *P*. *damicornis* in the *Artemia* only treatments ingested 120.6 ± 17.5 *Artemia* per 200 polyps h^−1^ at ambient temperature and 73.3 ± 17.3 *Artemia* per 200 polyps h^−1^ after thermal stress. In the MP & *Artemia* treatments, *P*. *damicornis* ingested 134.2 ± 11.9 *Artemia* per 200 polyps h^−1^ at ambient temperature and 111.3 ± 17.7 *Artemia* per 200 polyps h^−1^ after thermal stress (Fig. 2D).

## Discussion

In this study we investigated how microplastics ingestion and heterotrophy are impacted after thermal stress in two reef-building corals, *Montipora capitata* and *Pocillopora damicornis*. Our results revealed that prior exposure to thermal stress did not affect microplastics ingestion but can lead to decreased feeding on prey. We also found that ingesting microplastics did not affect the amount of prey ingested, and that these corals did not selectively ingest microplastics as has been observed in another species^33^. Additionally, we observed considerable variability in microplastics and *Artemia* ingestion rates under different scenarios between the two studied species. Our results suggest that coral species will respond differently to microplastics pollution following thermal stress events.

In contrast to previous studies^14,23^, we did not observe higher feeding rates in corals following thermal stress and subsequent bleaching. On the contrary, *Artemia* feeding rates significantly decreased for both species, and microplastics ingestion rates decreased slightly in *P*. *damicornis*. This may be due to the corals being stressed, which has been suggested to cause decreased tentacle activity and/or nematocyst function^22,24,34^. The fact that feeding did not increase may be due to not reaching a “bleaching threshold” needed to see an increased feeding response. In the present study, we followed a similar thermal stress regime to that of Grottoli *et al*.^14^ and, though we did not quantify bleaching (symbiont counts, pigment concentrations, photophysiology), most coral fragments were completely white, and the rest were very pale. Alternatively, it may be that energy reserve status is the mechanism controlling heterotrophic plasticity. In our study we measured ingestion immediately after thermal stress, whereas Grottoli *et al*.^14^ measured it two weeks after thermal stress exposure. Thus it may be that these corals needed to spend more time bleached in order to reach such critical energy levels and increase heterotrophy. Two corals, *Turbinaria reniformis* and *Galaxea fascicularis*, display increased feeding rates in as little as five days of exposure to thermal stress^22^ suggesting that such critical thresholds can be met rapidly, and supporting that this response is considerably variable among species. Furthermore, heterotrophic plasticity can also be driven by other environmental factors such as ultraviolet radiation and seasonal weather patterns^24,35^. With the increasing threat of thermal stress events and microplastics accumulation in the oceans, further research should compare feeding rates of prey (and microplastics), as well as assimilation and allocation of heterotrophic carbon to the corals’ energy reserves (e.g. lipids, carbohydrates). Feeding rates (prey/microplastics) and carbon transfer should be evaluated at several periods over the entire bleaching cycle, from the initiation of thermal stress to the full recovery of the coral.

Even though exposure to microplastics led to them being ingested by both species, it did not affect *Artemia* ingestion rates as expected. A similar behavior was observed in *A*. *poculata* which, following exposure to microplastics, did not change the amount it fed on live *Artemia* and copepods^32^. While this suggests that ingesting microplastics may not have a large effect on heterotrophic energy acquisition for these species, more information is needed to draw such a conclusion. First, this study was limited by the short duration (1 h) of feeding trials. For many corals that generally feed all night, constant exposure could allow microplastics to accumulate in the polyps and prevent further ingestion of prey. However, the amount of accumulation that occurs depends on retention time and egestion rates, which were not measured in this study. To our knowledge, there is currently no published information on accumulation rates (e.g. mass balance) in coral polyps constantly exposed to microplastics and should be a priority for future research. Additionally, we lack data on the assimilation rate of carbon and nutrients from the *Artemia* prey in corals exposed to microplastics. Though microplastics did not appear to act as a barrier to prey ingestion, at least in the short-term, they may act as a barrier to digestion and nutrient assimilation. In the oyster, *Pinctada margaritifera*, microplastics exposure did not affect ingestion rate but did significantly decrease macroalgae assimilation efficiency^36^.

Chemoreception did not appear to drive microplastics ingestion for either species studied here. In contrast, the presence of *Artemia* prey appeared to strongly influence whether microplastics were ingested for *P*. *damicornis*, which did not selectivity ingest microplastics. Allen *et al*.^33^ found that *A*. *poculata* ingested clean weathered plastics over bio-fouled ones and suggested that microplastics ingestion by corals was driven by phagostimulant (feeding cue) release by the plastics. However, in the present study, microplastics ingestion by *P*. *damicornis* was absent or negligible when exposed only to clean microplastics. In a similar study, symbiotic sea anemones, *Aiptasia pallida*, were also reluctant to ingest any microplastics, including nylon, polyester and polypropylene fibers, in the absence of prey tissue^37^. Differences among studies could be due to species specific responses to phagostimulants in plastics, and/or the use of different types of microplastics. The microplastics used in our study were all polyethylene, whereas Allen *et al*. (2017) used a mixture of plastic particles consisting of two-thirds polyethylene and one-third polystyrene, and it might be that only polystyrene released a phagostimulant. Further research should focus on the potential of phagostimulant release by different types of plastic. Our results suggest that chemicals released by certain plastics may drive selectivity in some corals, such as *A*. *poculata*, but not so in other corals, such as *P*. *damicornis*, that are simply at risk of inadvertently ingesting microplastics during times when they are feeding.

The fact that acute thermal stress led to decreased microplastics ingestion in this study does not eliminate corals’ risk of exposure, as microplastics were still ingested. The act of ingesting and then egesting microplastics is assumed to be energetically costly^7–9^, although further research is needed to determine how costly those behaviors are^38^. Additionally, exposure to microplastics can trigger rejection mechanisms, similar to how corals handle sediment exposure^39,40^, that also consume the coral’s energy reserves^7^. In other benthic marine invertebrates, microplastics ingestion has also led to weight loss^28^ and decreased fitness^29^ which were attributed to decreased prey ingestion or assimilation efficiency due to the presence of microplastics in the gut. In corals, chronic exposure to microplastics resulted in species-specific stress responses, including decreased growth, supporting the notion of depleted energy reserves^10^. Future research should focus on examining how and to what degree microplastics exposure and ingestion can affect a coral’s energetic status in the long-term, especially during bleaching when energy reserves are critical for the coral’s survival^41^. Furthermore, the ability of some corals to increase feeding due to bleaching or other factors could exacerbate these effects if microplastics ingestion increases accordingly, but this still needs to be determined. For future corals that will have to endure increasingly prolonged and intense thermal stress, and numerous other stressors^4^, any amount of energy wasted could be significant.

Results from this study, and from other studies that investigated microplastics ingestion by corals, support that some corals are likely more at risk of microplastics exposure than others^10^. For example, in agreement with Reichert *et al*. (2018), this study observed variable microplastics ingestion rates among coral species, and challenging Allen *et al*. (2017), our work showed that plastics are not so “tasty” to all corals. Furthermore, coral feeding rates can vary depending on a variety of factors^14,22–24,35^ and may potentially affect microplastics ingestion. Given the various responses to microplastics and feeding behaviors of corals, future research should focus on how, and which, corals are likely to be affected by microplastics under future scenarios.

Here we present the first study to examine the roles of thermal stress on microplastic ingestion and of microplastics exposure on heterotrophy in two reef-building corals. Overall, *P*. *damicornis* ingested more microplastics and fed more heavily on *Artemia* than *M*. *capitata*, while both species displayed decreased feeding on *Artemia* under thermal stress. When offered *Artemia*, *P*. *damicornis* readily ingested microplastics, but without live prey it ingested virtually no microplastics, indicating that chemoreception does not drive microplastics ingestion in all corals. Collectively, these results suggest that some coral species may be at greater risk of microplastics exposure than others. Further research should focus on the physiological effects of microplastics, how a corals’ feeding behavior influences its potential to ingest microplastics, how ingesting microplastics affect nutrient assimilation, which plastics release phagostimulants, and which coral species are affected by these phagostimulants. When used in the context of global change, these data will be critical for predicting the potential impact of microplastics on future corals and coral reefs.

## Methods

### Location and species

This study was conducted from June 21 to August 20, 2018, at the Hawai’i Institute of Marine Biology (HIMB), located in Kane’ohe Bay, O’ahu, Hawai’i (21.4282° N, 157.7919° W). We performed our experiments on two locally common reef-building coral species. *Montipora capitata* (rice coral) is a dominant reef-builder in Hawai’i. It was chosen for this experiment because it displays heterotrophic plasticity (increased feeding) following bleaching due to thermal stress^14,23^. This species occurs in plating and branching forms though only the branching form was used in this experiment. *M*. *capitata* is a small polyp species (ca. 0.8 mm diameter), has a perforate skeleton and has a plocoid coralite arrangement. *Pocillopora damicornis* (cauliflower coral) is a less-dominant branching coral species on Hawaiian reefs but is locally abundant^42^. To our knowledge, heterotrophic plasticity following thermal stress had not been reported for this species, which allowed us to investigate whether it also employs this strategy. *P*. *damicornis* has small polyps (ca. 1 mm diameter), a plocoid coralite arrangement and an imperforate skeleton.

### Experimental set-up

Ten colonies of *M*. *capitata* and *P*. *damicornis* (ca. 14 cm in diameter) were collected from 1–2 m depth in the inner lagoon surrounding HIMB on June 21 and July 12, 2018, respectively (DAR Special Activities Permit No. 2019–21). Colonies were collected at least 5 m apart to reduce the likelihood of getting genetically identical clones. From each colony, eight fragments (ca. 5 cm) were removed, attached to ceramic tiles and allowed to acclimate in an outdoor flow-through tank for 6–7 days. All tanks, one for acclimation and three for each temperature, were maintained with a volume of 400 L of sand-filtered seawater from Kane’ohe Bay and shaded to mimic photosynthetically active radiation (PAR) on the reef. Mean daytime PAR was 235 µmol photons m^−2^ s^−1^ and mean PAR at 12:00 was 522 µmol photons m^−2^ s^−1^ (Odyssey Submersible PAR Logger, Dataflow Systems LTD.). The average temperature of ambient seawater supplied during the acclimation period was 27.3 ± 0.5 °C, measured hourly (HOBO pendant temperature loggers #UA-002-64, Onset Computer Corporation).

After the acclimation period, four fragments from each colony were moved to ambient temperature treatments (27.2 ± 0.5 °C) and the other four were moved to increased temperature treatments (see below). The coral fragments were randomly assigned to one of the three tanks for each temperature treatment, and rotated weekly between tanks to minimize potential tank effects. For *M*. *capitata*, the temperature was increased slowly over five days to 30.8 ± 0.8 °C, similar to Palardy *et al*.^23^. In a preliminary experiment, *P*. *damicornis* experienced ca. 50% mortality under 30.8 °C so the water temperature was increased to only 29.2 ± 0.4 °C over five days. For both species, the increased temperature treatments lasted for 20 days and noticeable bleaching was observed, although not quantified. After the temperature treatment, the heaters were turned off and feeding trials began the following day, based on the assumption that *M*. *capitata* would increase feeding following thermal stress and bleaching^14,23^.

### Feeding trials

Feeding chambers were constructed of rectangular polycarbonate 3.7 L food pans fit with an adjustable circulation pump (Hydor pico 70, Hydor USA Inc.) on the lowest flow setting (49 L h^−1^) and an air-stone. The circulation pump was glued to the floor of one end of the chamber and the nozzle was pointed up at a 45° angle towards the middle of the chamber to break the water’s surface tension. It was necessary to supply air bubbles in the chamber to facilitate microplastics suspension in the water. Thirty minutes prior to adding the coral fragments, the chambers were filled with 2 L of 1 µm filtered seawater (FSW) and placed in water baths at ambient seawater temperature.

Each night, for ten consecutive nights, feeding trials were performed with all eight fragments from each colony (four fragments previously exposed to ambient temperature and four fragments previously exposed to increased temperature). The experiments started on July 20 for *M*. *capitata* and Aug. 11 for *P*. *damicornis*. The fragments from each temperature treatment were given one of four feeding treatments: (i) microplastics (2 particles mL^−1^) only, (ii) *Artemia* nauplii (2 individuals mL^−1^) only, (iii) microplastics (2 particles mL^−1^) and *Artemia* nauplii (2 individuals mL^−1^), and (iv) 1 µm FSW control. The concentration of microplastics used in this study was higher than what has been reported for coral refs. ^43,44^ but was lower than most previous experiments that studied microplastics ingestion by corals^7,8,33,45^. A high concentration of *Artemia* was also used because, as noted in previous studies^22,23^, it allowed for smaller sample sizes, minimized dissection time and increased statistical power. Green fluorescent polyethylene (confirmed by Fourier Transform Infrared Spectroscopy, see Supplementary Fig. S1) microbeads (Cospheric LLC.) with a diameter of 150–180 µm and a density of 1.025 g mL^−1^ were used for the microplastics treatment because they had similar mass (2.4 µg per particle) to the *Artemia* used and the same density as sea water. The microbeads were served clean (not bio-fouled) to allow for potential chemical cues to influence the corals’ feeding behavior^33^. Freshly hatched *Artemia* nauplii (Grade A, SLU strain, Brine Shrimp Direct, Ogden, UT; dry weight = 2.42 µg per individual^46^) were used because they fall within the prey size range for *P damicornis* and *M, capitata*^23,47^, and to facilitate the quantification of treatment concentrations and ingestion rates. FSW controls were used to account for the potential ingestion of residual microplastics that stuck to chamber components despite rigorous cleaning between trials. No microplastics were found in dissected control corals, thus control data were left out of further analyses.

Coral fragments were placed in the feeding chambers each day at 12:00 h to give them ample time to acclimate to the chamber and digest any previously ingested prey^23^. At 20:00 h, microplastics and *Artemia* nauplii were added to the feeding chambers and the coral fragments were allowed to feed for one hour before being removed from their chambers and fixed immediately in 10% formalin. Though both species used in this study presumably feed throughout the night, previous research has shown that they feed heavily enough within the first hour of dusk to draw meaningful biological conclusions^23,48^, thus a one hour feeding duration was used. The next day, the number of microplastics and *Artemia* ingested by 200 polyps were counted by dissecting 200 polyps from each fragment under a stereo microscope (10–40x) using fine dissection probes, forceps and a UV light. An ingestion was defined as a microplastic or *Artemia* nauplii found in the coral polyp and did not include any information on egested particles. Microplastics counted in the polyps were nudged sufficiently to be certain that we were counting the green fluorescent microbeads and not autofluorescence of the coral or symbionts. Microplastics and *Artemia* ingestion rates are reported as the mean number of ingestions per 200 polyps h^−1^ ± one standard error.

### Statistics

Microplastic and *Artemia* ingestion were compared separately for each species (n = 10) following a fully factorial 2 × 3 (2 temperatures × 3 feeding treatments) mixed effects permutation analysis of variance, using the aovp function^49^ in R (Rstudio v.1.1.463). This randomization procedure was used because most of the data did not meet the normality nor the equal variance assumptions of a typical ANOVA, due to high occurrences of zeros in the data (no ingestions by corals in some treatments). Temperature and feeding treatments were treated as main effects and colony as a random effect. The aovp function was ran with 10,000 iterations and results were considered significant when p < 0.05.

## Supplementary information

Supplementary Information

## Data Availability

The datasets generated during and analysed during the current study are available in the Figshare repository, 10.6084/m9.figshare.8264084.v1.
