# Supplementary material for: Microplastics ingestion and heterotrophy in thermally stressed corals
Source: Sci Rep. 2019 Dec 3;9:18193. doi: 10.1038/s41598-019-54698-7 (PMC6890796; doi:10.1038/s41598-019-54698-7)
Supplement: Supplementary file 1 — Supplementary Information [file 41598_2019_54698_MOESM1_ESM.docx]

**Microplastics ingestion and heterotrophy in thermally stressed corals**

Jeremy B. Axworthy^1^* and Jacqueline L. Padilla-Gamiño^1^

^1^School of Aquatic and Fishery Sciences

University of Washington

Seattle, Washington 98195, USA

*Correspondence: jeremyax@uw.edu


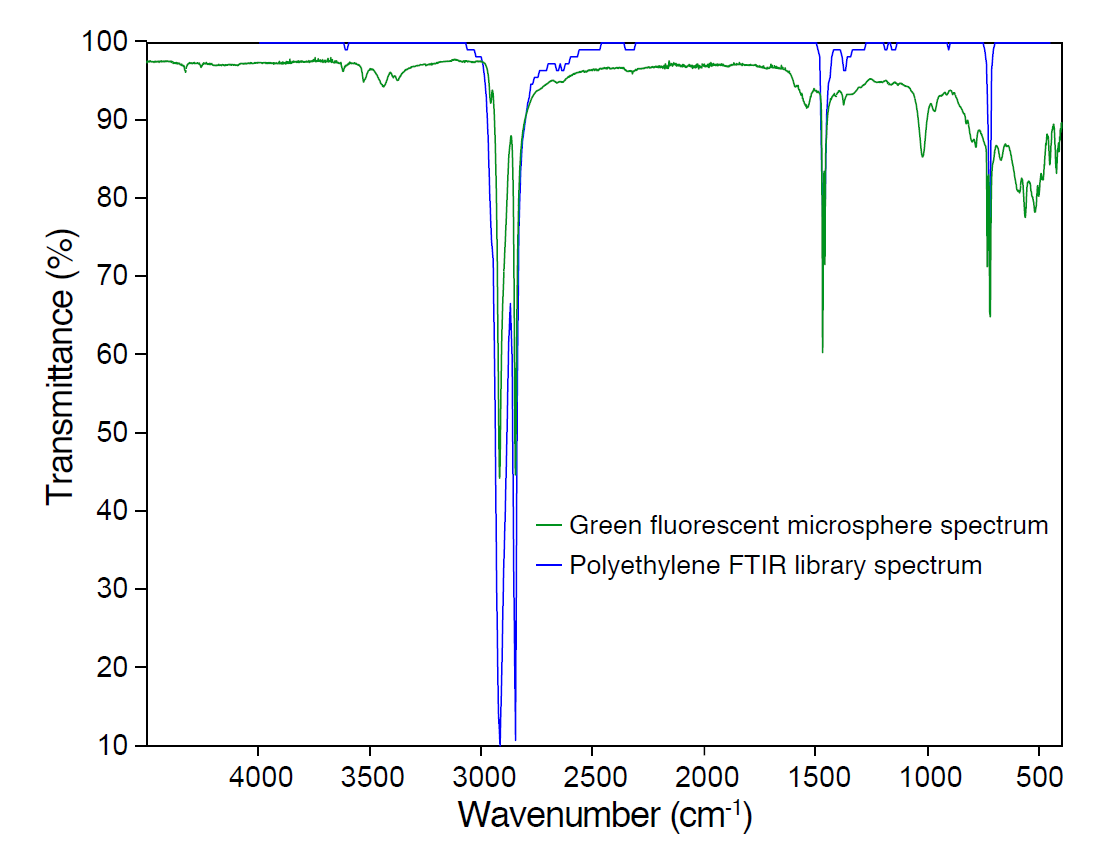


Figure S1: Polymer confirmation of experimental microplastics by Fourier Transform Infrared Spectroscopy (FTIR).
